# Genetic Primary Microcephalies: When Centrosome Dysfunction Dictates Brain and Body Size

**DOI:** 10.3390/cells12131807

**Published:** 2023-07-07

**Authors:** Sarah Farcy, Hassina Hachour, Nadia Bahi-Buisson, Sandrine Passemard

**Affiliations:** 1 UMR144, Institut Curie, 75005 Paris, France; sarah.farcy@curie.fr; 2 Inserm UMR-S 1163, Institut Imagine, 75015 Paris, France; 3 Service de Neurologie Pédiatrique, DMU INOV-RDB, APHP, Hôpital Robert Debré, 75019 Paris, France; hassina.hachour@aphp.fr; 4 Service de Neurologie Pédiatrique, DMU MICADO, APHP, Hôpital Necker Enfants Malades, 75015 Paris, France; nadia.bahi-buisson@aphp.fr; 5 Université Paris Cité, Inserm UMR-S 1163, Institut Imagine, 75015 Paris, France; 6 Université Paris Cité, Inserm UMR 1141, NeuroDiderot, 75019 Paris, France

**Keywords:** microcephalic dwarfism, centrosome, primary microcephalies, MCPH, brain development disorders, neural progenitors division

## Abstract

Primary microcephalies (PMs) are defects in brain growth that are detectable at or before birth and are responsible for neurodevelopmental disorders. Most are caused by biallelic or, more rarely, dominant mutations in one of the likely hundreds of genes encoding PM proteins, i.e., ubiquitous centrosome or microtubule-associated proteins required for the division of neural progenitor cells in the embryonic brain. Here, we provide an overview of the different types of PMs, i.e., isolated PMs with or without malformations of cortical development and PMs associated with short stature (microcephalic dwarfism) or sensorineural disorders. We present an overview of the genetic, developmental, neurological, and cognitive aspects characterizing the most representative PMs. The analysis of phenotypic similarities and differences among patients has led scientists to elucidate the roles of these PM proteins in humans. Phenotypic similarities indicate possible redundant functions of a few of these proteins, such as ASPM and WDR62, which play roles only in determining brain size and structure. However, the protein pericentrin (PCNT) is equally required for determining brain and body size. Other PM proteins perform both functions, albeit to different degrees. Finally, by comparing phenotypes, we considered the interrelationships among these proteins.

## 1. Introduction

Microcephaly is a brain growth defect responsible for a spectrum of neurodevelopmental disorders [1]. Microcephaly identified before birth is congenital and called primary microcephaly (PM), whereas secondary microcephaly is postnatal. This old group of diseases was known only to child neurologists and neuropathologists who had no other etiology to propose at the other time than in utero maternofetal infections to explain this disorder; however, this topic attracted the attention of researchers in the late 1990s–early 2000s, when it was found that this disorder has a genetic origin as well.

The first mutations identified in “microcephaly genes” are responsible for causing congenital, isolated, or primary microcephaly with recessive inheritance, and are termed microcephaly primary hereditary, or MCPH (MCPH1, OMIM # 251200 (*MCPH1* [2]), MCPH2, OMIM # 604317 (*WDR62* [3]), MCPH3, OMIM # 604804 (*CDK5RAP2* [4,5]), MCPH4, OMIM # 604321 (*CASC5* [5]) and MCPH5, OMIM #608716 (*ASPM* [6])). The original OMIM classification continues to reference newly identified PM genes included under the term “MCPH” (30 “*MCPHs* genes”) until now. However, the phrase “genetic PMs” gained precedence over “MCPH” as new-generation sequencing approaches demonstrated that variants in the same gene could cause several diseases, such as: (i) MCPH and microcephalic primordial dwarfism (MPD) [4,7,8,9], (ii) MCPH and malformations of cortical development (MCDs) [10,11], and (iii) MCPH and neurosensory disorders [4,12]. Considering genotypes and phenotypes together, all these defects, owing mainly to their recessive inheritance, were grouped under the term “genetic PMs”.

Information related to these microcephaly-causing genes and their functions has opened new research avenues in the genetics of species evolution, neuroscience, and developmental biology. This information has helped elucidate a critical step in brain growth, i.e., embryonic neurogenesis [13,14,15]. Significant advances in cell biology approaches have made it possible to identify the mechanisms underlying the origin of PMs. Over the last 20 years, many teams have contributed to deciphering the proteins and organelles involved in cell division and proliferation (see recent reviews [16,17,18,19,20,21,22]). Excellent reviews have summarized the impact of the defects of one of the thousands of proteins that constitute the mitotic spindle on cell division and survival [23,24,25,26,27]).

The biology of PM is a field that involves crosstalk among four fields of expertise, i.e., genetics, neurology, neurosciences, and cell biology. Therefore, the study of PM biology and treatment has been approached from one of those angles. Here, we provide an overview of the current knowledge of neurological and genetic approaches in the microcephaly field to understand this fascinating issue. Although they belong to the same system, that is the mitotic spindle pole and furrow, why does the absence or presence of an abnormal centrosome or spindle protein have different phenotypic consequences in vivo? By reviewing recent studies on the cellular and molecular mechanisms of the most iconic PMs, we will discuss those that only affect brain growth or brain and body growth in humans and those that most impact the cognition of affected individuals.

## 2. Primary Microcephaly: Small Brain Size or Small Brain and Body Sizes?

### 2.1. Primary Microcephaly: An Early Defect in Brain Growth with or without Cortical Malformations

**Figure 1:**
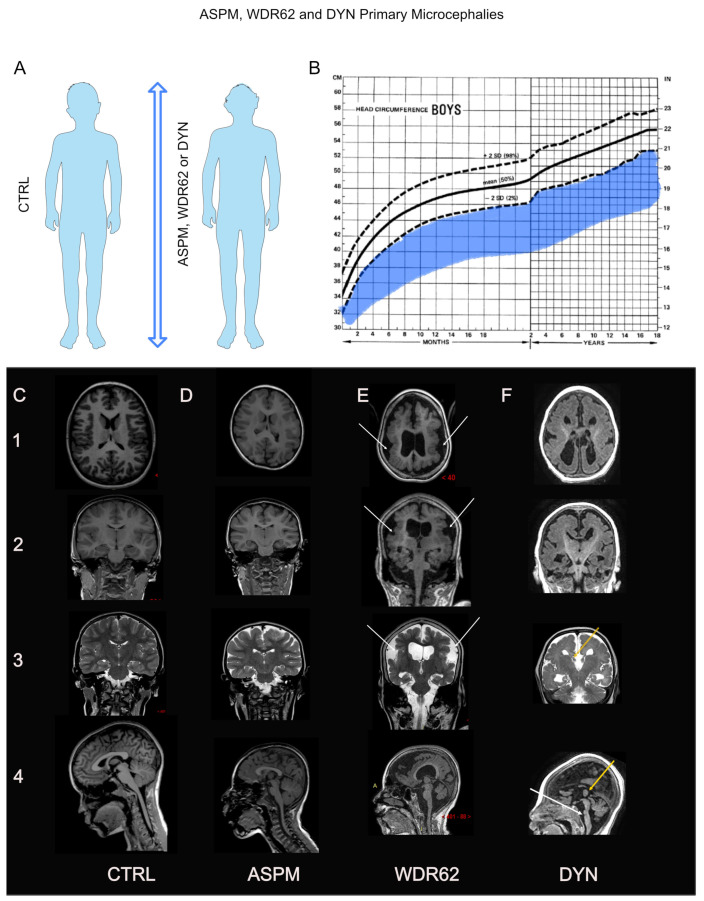
Body size, brain growth evolution, and structural neuroanatomy of ASPM-, WDR62-, and DYNC1H1-related primary microcephalies. (**A**): Schema illustrating the most emblematic primary microcephalies (PMs) caused by *ASPM*, *WDR62*, or *DYMC1H1* mutations, characterized by a reduction in brain volume without reduction in body size. (**B**): Occipitofrontal head circumference (OFC) showing that brain growth in individuals with *ASPM*, *WDR62*, or *DYMC1H1* PM (in blue) is mainly below the normal range, taking age and sex into account. (**C**–**F**): Brain magnetic resonance imaging (MRI) of healthy control (**C**) as compared to patients with primary PM caused by *ASPM* (**D**), *WDR62* (**E**), or *DYNC1H1* (**F**) mutations. From top to bottom (1–4): Axial T1, coronal T1 (or axial and coronal T2 FLAIR for (**F**), coronal T2, and sagittal T1-weighted images illustrating the main cortical malformations associated with PMs caused by mutations in *ASPM*, *WDR62*, and *DYNC1H1*. Reduction in brain volume and gyral simplification is evident in all cases. Bilateral polymicrogyria is observed frequently in patients with *WDR62* mutations ((**E**), white arrow). Corpus callosum agenesis (F3–4, yellow arrows), pachygyria (F1–3), and brainstem hypoplasia (white arrows, F4) are frequently associated with *DYNC1H1* mutations.

Microcephaly is a frequent defect in brain growth that affects 2–3% of the population worldwide [28]. It is defined by physicians based on occipitofrontal head circumference (OFC) below the normal range (<−2 standard deviation (SD)) based on age and sex. Congenital microcephaly or PM is detected before or at least at birth and is considered severe when the deviation from the mean normal OFC is below –3 SD. The prevalence of severe PM is 0.5–1 per 1000 live births. The defect is often detected in the second trimester of pregnancy using high-performance ultrasound, indicating that the slowdown of brain growth occurs early in the first trimester at the peak of neurogenesis in humans. In addition, intrauterine growth retardation may be associated with microcephaly at birth. However, length and weight are usually compensated within the first 24 months of life. Unlike weight and height, the kinetics of embryonic and postnatal brain growth in individuals with PM typically worsen with age until adulthood (Figure 1), suggesting that the later stages of brain development, i.e., astrocytogenesis, myelination, and synaptogenesis, may also be affected (see [29,30]).

Cortical malformations such as pachygyria (thick cerebral cortex), polymicrogyria (small, irregular, and shallow cerebral gyri), schizencephaly (cortical mantle interruption), and periventricular nodular/subcortical neuronal heterotopia or lissencephaly (smooth brain) may be associated with PMs [31]. Examples of MCDs often associated with PMs are shown in Figure 1. In addition to microcephaly, these MCDs attest to critical-staged impairments in neurogenesis and neuronal migration. These MCDs severely affect the motor and intellectual prognoses in patients with PMs and cause epilepsy.

### 2.2. Primary Microcephaly Is an Early Defect in Brain Growth with or without Defects in Body Growth (Microcephalic Primordial Dwarfism)

**Figure 2:**
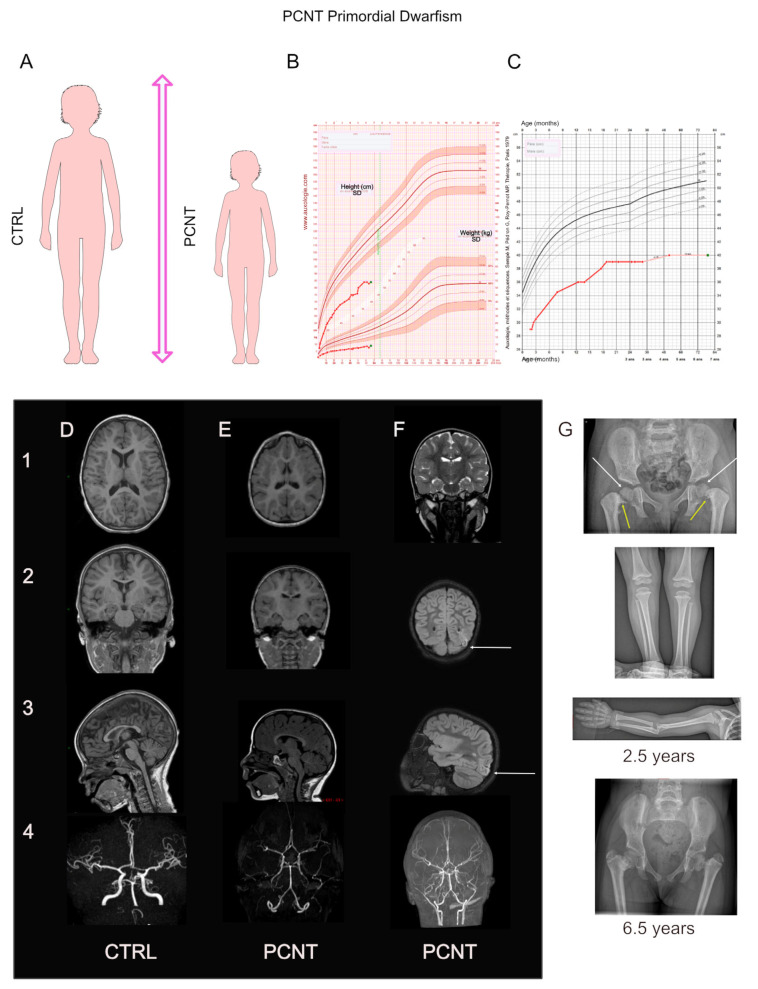
Stature, brain growth evolution, and structural neuroanatomy in patients with primordial dwarfism (MPD) due to *PCNT* mutations. (**A**): Schema illustrating PCNT primordial dwarfism characterized by a proportionate reduction in both brain size and body size. (**B**): Height (top) and weight (bottom) growth curve of a girl carrying *PCNT* mutations with a deviation of −7.5 SD from the mean normal for weight and height. (**C**): The OFC growth curve of a girl carrying *PCNT* mutations with a deviation of −8 SD from the mean. (**D**–**F**): Brain MRI (1–3) and magnetic resonance angiography (MRA, 4) of healthy control (**D**), as compared to the same 6.5 year-old girl carrying PCNT mutations (**E**,**F**). Axial T1 (1 (**D**,**E**)), coronal T1 (2 (**D**,**E**)), sagittal T1-weighted images (3 (**D**,**E**)), coronal T2 (1 (**F**)), and coronal and sagittal T2 FLAIR-weighted images (2–3, (**F**)) illustrating brain structure and vascular complications related to *PCNT* mutations. Note the left occipital cortico-subcortical lesion secondary to sequelae from an ischemic stroke (2–3 (**F**), white arrow) due to a tight stenosis at the origin of the left posterior cerebral artery. This patient exhibited the typical arteriopathy related to *PCNT* mutations: (i) a bilateral tight and long stenosis of the extracranial internal carotid arteries from the carotid bifurcation, with neovascularization, and (ii) a narrowing and rigid appearance of intracranial internal carotid arteries, circle of Willis, vertebral and basilar arteries. (**G**): Skeletal complications in PCNT primordial dwarfism, including bilateral coxa vara, narrow ischia, irregular metaphysis (yellow arrows), bilateral proximal femoral epiphyseolysis (white arrows), and brachymesophalangia of the Vth fingers.

In the early 1960s, Helmut P.G. Seckel, Prof. of Pediatrics at the University of Chicago, described individuals with a rare phenotype of proportionate short stature or primordial dwarfism, associated with an extreme smallness of the head, with a peculiar aspect resembling “bird-headed” (Figure 2). Prof. Seckel afforded his name to this rare defect, which is diagnosed based on the following five criteria: (1) extreme statural dwarfism (adult standing height of ~120 cm) and in children, a deviation of −6 to −8 SD from the mean normal standing height or length; (2) a head circumference of 39–42 cm in adults (normal: >52 cm) and ~27 cm in newborns; (3) a proportionate smallness of skull and face; (4) a degree of intellectual disability; and (5) a frequent presence of congenital malformations, in addition to the abnormal bird-like facies [32]. In the 1990s, the term “Seckel syndrome” (OMIM **#** 210600) was expanded to include other clinical types of MPDs, such as Majeski type 2 syndrome (or osteodysplastic primordial dwarfism type 2 (MOPD2) [33]) and Meier–Gorlin syndrome [34]. The progressive identification of the molecular cause of each syndrome has led physicians to propose a new clinicopathological classification for MPDs that considers their phenotypes and genotypes, which also facilitates the description of new entities (see Box 1). These effects are inherited in a recessive manner.

Box 1 **Microcephalic primordial dwarfism (MPD).**

*Definition*:

Severe pre- and postnatal growth failure is associated with proportionate or disproportionate microcephaly, with an autosomal recessive inheritance.

*Included syndromes*:

**Seckel syndrome**: Intrauterine and postnatal growth retardation, proportionate microcephaly with intellectual disability, and a characteristic “bird-headed” facial appearance [32]. Seckel syndrome PMDs are caused by biallelic variants in *ATR*, *RBBP8*, *CENPJ*, *CEP152*, *CEP63*, *NIN*, *DNA2*, *TRAIP*, and *NSMCE2* genes.

**Osteodysplastic primordial dwarfism type 2** (MOPD2): Severe intrauterine growth retardation with proportionate microcephaly, mesomelia (shortness of the middle portion of a limb), skeletal dysplasia, abnormal dentition, insulin resistance, and cerebral vascular disease with progressive stenosis and occlusion of the cerebral arteries and moyamoya disease. MOPD2 is caused by biallelic variants in the *PCNT* gene [35,36].

**Meier–Gorlin syndrome**: Severe intrauterine and postnatal growth retardation, microcephaly, bilateral microtia (underdevelopment of the external ear), and aplasia or hypoplasia of the patellae. Meier–Gorlin syndrome MPDs are caused by biallelic variants in *ORC1*, *ORC4*, *ORC6*, *CDT1*, *CDC6*, *GMNN*, *CDC45L*, and *MCM5* genes [37].

**Bloom syndrome**: Severe intrauterine and postnatal growth retardation, severe microcephaly, immunodeficiency, sensitivity to sunlight, insulin resistance, and a high risk of cancers with multiple types and sites at an early age. Cognitive abilities seem to be more preserved than in other MPDs. Bloom syndrome is caused by biallelic variants in *RECQL3/BLM* gene [38].

**Ligase IV deficiency**: Severe dwarfism with severe and disproportionate microcephaly, combined immunodeficiency, sensitivity to ionizing radiation, and predisposition to cancer. A neurodevelopmental delay seems expected in this syndrome. Ligase IV deficiency is caused by biallelic variants in the *LIG4* gene [39].

***XRCC4* deficiency**: Severe intrauterine and postnatal growth retardation similar to *LIG4* deficiency, extreme microcephaly, sensitivity to ionizing radiation without tumor or immunodeficiency in affected children is reported. *XRCC4* deficiency is caused by biallelic variants in the *XRCC4* gene [40].

### 2.3. Primary Microcephaly: An Early Defect in Brain Growth with or without Neurosensory Disorders

In addition to microcephaly with or without primordial dwarfism, individuals with PM may exhibit neurosensory disorders (Box 2). These include visual and hearing impairments rarely present at birth and usually occurring during the first few years of life. It is hard to affirm that this neurosensory impairment is caused by variants in PM genes and not due to an undercurrent environmental cause, mainly infectious embryo-fetopathy. Consequently, clinicians have to refer patients to experienced ophthalmologists and ENT specialists who can discriminate genetic from environmental causes.

Visual impairment associated with PM may be caused by anomalies in the development of: (i) the anterior segment of the eye, i.e., microphthalmia, microcornea, and cataracts and (ii) posterior segment, i.e., the abnormal pigmentation of the retina, chorioretinopathies with chorioretinal lacunae and retinal folds, leading to retinal detachment or cone-rod retinal dystrophy. Optic nerve hypoplasia has also been previously reported [41].

Sensorineural hearing loss may be associated with PM. However, as this congenital sensory disorder is common and affects one in 500 newborns, environmental etiologies that account for half of all etiologies need to be ruled out first [42]. The remaining 50% of neurosensory hearing loss cases are of genetic origin and classified into either syndromic causes or isolated, i.e., non-syndromic, causes. Sensorineural hearing loss associated with PMs is syndromic, with inner-ear malformations observed in 40% of patients [42,43,44].

Box 2 **Primary microcephaly and neurosensory disorders.**

*Definition*:

Primary microcephaly is associated with chorioretinopathy or sensorineural hearing loss, or both.

*Included syndromes*:

**KIF11 PM**: Autosomal dominant microcephaly due to heterozygous *KIF11* variant characterized by (i) developmental ocular abnormalities, e.g., chorioretinopathy (choroidal atrophy and non-progressive dysplasia of the retina), retinal folds and detachment, microphthalmia (a developmental disorder of eyes abnormally small at birth), and myopic and hypermetropic astigmatism [45] and (ii) feet congenital lymphedema.

**TUBGCP4/6 PM**: Autosomal recessive microcephaly characterized by (i) chorioretinopathy similar to cone-rod retinal dystrophy due to biallelic variants in *TUBGCP6* [46] or (ii) chorioretinal dysplasia, with multiple punched-out retinal lesions, and other anomalies, e.g., microphthalmia, retinal folding, retinal detachment, optic nerve hypoplasia, the absence of retinal vessels, and round areas of chorioretinal atrophy due to biallelic *TUBGCP4* variants [47].

**PLK4 PM**: Autosomal recessive microcephaly due to biallelic *PLK4* variants characterized by (i) chorioretinopathy with pale optic discs, thin retinal vessels, bilateral macular atrophy, and severe generalized retinopathy, but also microphthalmia, microcornea, or cataract, and (ii) dwarfism [46] similar to Seckel syndrome [48].

**CDK5RAP2 PM**: Autosomal recessive microcephaly due to biallelic *CDK5RAP2* variants [4] characterized by (i) progressive hearing loss due to a specific cochlear malformation (small cochlea and a simplification of the cochlear spiral), (ii) ocular defects, including microphthalmia and retinal pigmentation defects, and (iii) interhypothalamic adhesion [12].

### 2.4. Primary Microcephaly: An Early Defect in Brain Growth with or without Intellectual Disability

The functional neurodevelopmental consequences of PMs include the intellectual disability (ID) of variable severity, behavioral disorders, epilepsy, neurosensory impairments, and cerebral palsy [12,30,49,50,51,52,53]. Identifying the impact of microcephaly on patients’ intellect is a central issue, as it is necessary to ascertain the degree of autonomy and future social insertion for these individuals. However, an accurate assessment of the intellectual abilities of these individuals is performed in rare cases, and motor and intellectual prognoses are delineated only for the most frequent forms [12,51,53]. There is a real benefit in precisely identifying the intellectual abilities of these patients to ascertain their autonomy. Moreover, identifying the PM type with the best or worst prognosis is also necessary as this informs the quality of neuronal and neuronoglial networks in these patients.

## 3. *ASPM*, *WDR62*, and *Dynein*: Three Emblematic PM Genes Implicated in PMs with or without MCDs

### 3.1. ASPM: Phenotype–Gene Relationships

#### 3.1.1. Genetics

**Figure 3:**
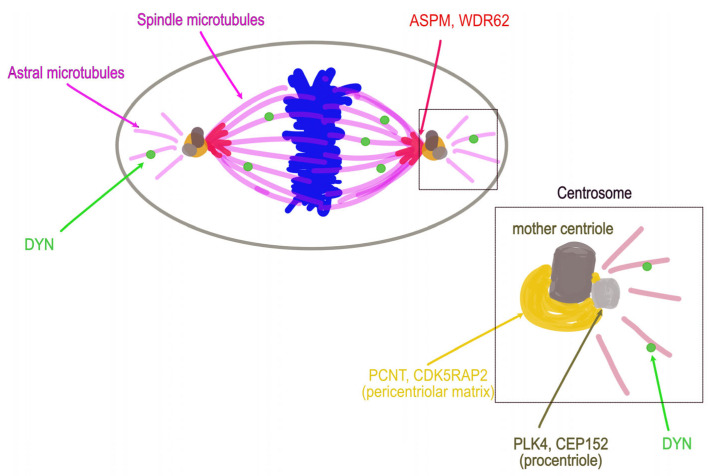
Localization of the most emblematic primary microcephaly proteins during mitosis. Scheme showing ASPM, WDR62, and DYNC1H1 at the mitotic spindle, PCNT and CDK5RAP2 at the pericentriolar matrix of the centrosome, and CEP152 and PLK4 at the centriole.

The *ASPM* (NM_018136.5) gene encodes the abnormal spindle-like microcephaly-associated protein, a minus-end microtubule-associated protein localized to spindle poles, necessary for spindle pole organization and orientation as it modulates microtubule dynamics at the centrosome and cytokinesis [54,55,56] (see Figure 3). *ASPM* is the most frequently mutated gene in autosomal recessive PMs. Since the identification of the first patients [6], 861 individuals from 390 families carrying 210 different biallelic variants spread over the gene have been reported (for synthesis, see [50,51] and more recently [57,58,59,60,61,62,63,64,65,66,67,68,69,70,71,72,73,74,75]). Approximately 55% of published cases were of Pakistani origin. Of the 210 variants, 207 were loss-of-function mutations or large deletions encompassing several exons of the *ASPM* gene, predicted to lead to the absence of the protein or a nonfunctional truncated protein. Two specific loss-of-function *ASPM* variants (c.9754del; pArg3252Glufs*10 [76], and c.9984+1G>T, predicting the removal of the intron 25 splice donor site [54]) have been shown to generate a truncated but stable ASPM protein with reduced expression at spindle poles [54].

#### 3.1.2. Growth

Retrospectively, in Europe, microcephaly in children carrying *ASPM* mutations was detected during pregnancy in 53% of cases, mainly during the third trimester [51]. Although intrauterine growth retardation was often observed, followed by a short stature within the first two years of life, body growth normalized as feeding difficulties disappeared and the children aged. The heights of these individuals in adulthood are usually comparable to those of healthy individuals [51], except when kyphoscoliosis occurs [60], which suggests that *ASPM* is not essential for body growth. 

#### 3.1.3. Brain Development and Cognition

The brain growth of these individuals is below the normal range before birth and typically slows postnatally (Figure 1). The brains of these children have long been considered “small-scaled” brains, with a characteristic simplification gyration. However, an analysis using structural brain imaging has highlighted that ASPM-PM is not a homothetic reduction in brain volume. The volume of the basal ganglia, cerebellum, and brain stem are better preserved than that of the cerebral cortex, and regional differences in volume also exist within the cerebral cortex. The neocortex is reduced by 50% in volume (and more in surface area, which is insufficiently compensated for by an increase in thickness). However, the hippocampal volume is nearly comparable to that of healthy individuals [77]. Cognitive assessment of these children showed retention of their mnesic abilities, concordant with their normal hippocampal volume. In parallel, a decrease was recorded in their intellectual abilities (mean full-scale intellectual quotient (FSIQ): 57.5 ± 10 SD, range 40–82, *n* = 40), resulting from a marked reduction in neocortical volume [77]. However, MCDs are rare under this condition, and polymicrogyria (an excessive number of abnormally small cerebral gyri with cortical overfolding) is the only reported MCD in ASPM-PM and has been identified in only four of ninety-seven patients who underwent brain MRI [51,78,79]. Epilepsy is much more frequent and affects 20% of patients with *ASPM* mutations in Europe [49,51]; thus, it is not necessarily caused by an MCD associated with ASPM-PM. A high proportion of this group of patients comes from consanguineous families. It remains unclear whether *ASPM* mutations alone cause epilepsy without MCD or whether additional variants in other PM- or epilepsy-causing genes exacerbate the phenotype, as shown by Duerincks et al. and Makhdoom [62,66], underlying variabilities between patients or siblings. Finally, although this question remains unaddressed, the loss of normal excitatory–inhibitory neuronal balance in the cerebral cortex of patients carrying *ASPM* mutations may also explain the high rate of epilepsy in patients without MCD. 

### 3.2. WDR62: Phenotype–Gene Relationships

#### 3.2.1. Genetics

The *WDR62* (NM_001083961.2) gene encodes the WDR repeat-containing protein 62, a minus-end microtubule-associated protein localized to spindle poles with roles in spindle assembly and orientation, centriole duplication, cilium disassembly, and thus in mitotic progression and neuronal migration [15,80,81,82,83] (see Figure 3). The *WDR62* gene is the second most frequent mutated gene in autosomal recessive PMs. Since the identification of the first patients [3,10,11], 156 individuals from 74 families, along with 66 different biallelic variants spread over the gene, have been reported (see synthesis in [53,84] and more recently [66,85]). Unlike *ASPM*, the missense and nonsense/frameshift variants of *WDR62* exhibited equal representation. However, the effects of these variants on protein function have not been studied. 

#### 3.2.2. Growth

Weight and height are normal in patients with *WDR62* mutations, suggesting that *WDR62* is not essential for body growth. 

#### 3.2.3. Brain Development and Cognition

Microcephaly is usually detected at birth, but a few newborns have normal OFC, which may explain the low rate of in utero diagnosis of this PM type despite careful ultrasound scan monitoring. The OFC declines with age and reaches a mean deviation of −6.5 SD from the mean normal OFC at adolescence [53,84]. In addition to the reduction in brain volume characteristic of PM, WDR62-PM is recognizable in routine brain MRI because of its association with MCDs (see Figure 1). These MCDs include pachygyria in 70% of cases resulting from gyral simplification with broad gyri and an abnormally thick cortex. However, relatively more severe MCDs, such as lissencephaly, bilateral schizencephaly (cortical mantel interruption), polymicrogyria, or neuronal heterotopia grouped in an abnormal location (along the ventricle or within the white matter) occur in 30% of affected individuals [3,10,11,53,86,87,88,89,90,91,92]. Fewer severe cortical malformations have been identified in patients with variants inside the WD-domain [53]. These severe MCDs are evidence of neuronal migration disorders and are almost invariably responsible for epilepsy [3,10,11,53,64,87,89,91,92,93,94,95,96,97,98]. It is difficult to understand the cognitive consequences of *WDR62* variants. Considering the associated cortical malformations, a poor functional prognosis can be expected. Although only a few individuals could have been subjected to cognitive evaluation, we were able to assess eleven children out of seventeen on international Wechsler scales, identifying that three of them had mild ID, four of them had moderate ID, and four had severe ID (mean FSIQ: 51.8 ± 12.6 SD, range 40–70) [53]. Remarkably, despite their mild-to-moderate ID, these patients have significant autonomy in daily life and adequate social interactions. It is important to note that one patient out of seventeen had signs of motor decline in the second decade of life, with the occurrence of ataxia and tremors raising fears of neuronal degeneration [53].

### 3.3. Dynein: Phenotype–Gene Relationships

#### 3.3.1. Genetics

*DYNC1H1* (NM_001376.5) encodes a part of the cytoplasmic dynein complex essential for retrograde cargo transport in axons and dendrites and is thus involved in neuronal development, morphology, and survival (see Figure 3). Since the first mutations were identified in patients with cortical malformations [99], 130 individuals have been reported to carry the majority of dominant missense variants in evolutionarily well-conserved domains with functional roles in processive and power-stroke movements [99,100,101,102,103,104,105,106,107,108,109,110,111,112,113,114,115,116,117,118,119,120,121].

#### 3.3.2. Growth

Weight and height are normal in DYNC1H1-related disorders or dyneinopathy, suggesting that *DYNC1H1* is not essential for body growth.

#### 3.3.3. Brain Development and Cognition

DYNC1H1-related disorders or dyneinopathy encompasses a spectrum of overlapping disorders, ranging from exclusive neuromuscular phenotype or DYNC1H1–NMD, or peripheral dyneinopathy, to combined neuromuscular and central nervous system dyneinopathy, on either side of the spectrum [121]. Nearly half of the reported patients show exclusive peripheral dyneinopathy predominantly involving the lower limbs, termed spinal muscular atrophy with lower-end predominance [113,122], exhibiting delayed motor milestones, muscle weakness, atrophy hyporeflexia, and skeletal limb abnormalities. These patients do not present the disrupted brain structure or function as is observed in microcephaly, intellectual disability, or cortical malformations. *DYNC1H1* mutations causing this phenotype are usually located in the tail domain of *DYNC1H1* (53AA–1867AA), predominantly within the dimerization domain (300AA–1140AA). Previous studies have demonstrated that these tail domain mutations do not disrupt the retrograde movement of dynein along microtubules, in contrast to motor domain mutations. Instead, they shorten the run lengths of the processive dynein–dynactin–BICD2N complexes, possibly disrupting neuronal cargo delivery [123]. 

A second group of patients with central and peripheral dyneinopathy demonstrated a complex phenotype combining predominant lower extremity muscle atrophy and a variable degree of intellectual and global developmental delay, brain malformations in MRI, or both. Patients with central dyneinopathy or central and peripheral dyneinopathy show different degrees of ID, with more severe phenotypes causing epilepsy or spastic paraplegia in affected individuals. Microcephaly usually is detected at birth in these patients; however, a few newborns exhibit normal OFC. Microcephaly is associated with severe cortical malformations and correlates with the severity of MCD. Contrary to other causes of PMs, OFC rarely deviates from the mean normal OFC below −4 SD (personal communication). In MRIs, these patients show a spectrum of MCDs, including pachygyria, dysgyria, and polymicrogyria (see Figure 1), frequently with ventricular anomalies, dysmorphic basal ganglia and corpus callosum, and cerebellar hypoplasia [102,119]. *DYNC1H1* mutations causing this phenotype are usually located in the motor domain, MTBD, or linker region, resulting in a disturbed motor activity, possibly secondarily to the severe disruption of neuronal migration and myelination.

## 4. PCNT: The Major Microcephalic Primordial Dwarfism-Causing Gene

### 4.1. PCNT: Phenotype–Gene Relationships

#### 4.1.1. Genetics

The *PCNT* (NM_006031.56) gene encodes pericentrin, a central component of the pericentriolar material (PCM) arranged around a pair of centrioles that constitute the centrosome [124,125,126,127,128,129] (see Figure 3). *PCNT* is the most frequently mutated gene in patients with recessive MPDs. Since the first patients were identified [35,36], 139 individuals from 116 families carrying 115 different biallelic variants spread over the gene have been reported [66,130,131,132,133,134,135,136,137,138,139,140,141,142,143,144,145,146,147,148,149,150,151,152,153]. All variants had loss-of-function mutations (nonsense, frameshift, and splicing). The effects of a few variants on protein function have also been studied. Homozygous (1887delA; S629fs), (3568_3569insT; C1190fs), (c.6329G>A; p.W2110X), and (c.6711delG; p.E2237fsX2244) variants resulted in the absence of the PCNT protein in the lymphoblastoid cell lines of patients [35,36], whereas the (c.658G>T; E220X) variant products were truncated proteins [36].

#### 4.1.2. Growth

Intrauterine growth retardation is a hallmark of this disorder, with deviations of −5.2 ± 1.9 SD and −4.3 ± 1.6 SD from the mean normal length at birth and the mean normal OFC, respectively. *PCNT* mutations cause MPD, specifically, MOPD2 or Seckel syndrome [35,36]. Based on this definition, growth retardation in both syndromes is proportional and affects the body and brain growth equally [154]. For a mean age of 8.3 years, the reported patients showed deviations of −8.4 ± 2.2 SD and −8.3 ± 2.4 SD from the mean normal height and the mean normal OFC, respectively (*n* = 89 and 81 measures for height and OFC, respectively). No significant difference was observed between height and OFC parameters (*t*-test) [35,36,130,132,135,136,137,138,140,141,142,143,144,145,146,149,150,151,155]. The weight of these patients was less affected than height and OFC (−6.4 ± 3 SD, *p* < 0.0001, one-way analysis of variance). Morphological features, including skeletal dysplasia in MOPD2 and Seckel syndrome, are described in Box 1 and depicted in Figure 2.

#### 4.1.3. Brain Development and Cognition

Microcephaly is severe (deviation of −8.3 ± 2.4 SD from the mean normal OFC, range: −4.6 to −15 SD for a mean age of 8.3 years) without a real disruption of cortical architecture, despite reports of pachygyria and gyral simplification in a few patients [130,140,143,144,145]. Polymicrogyria is rare (one in fifty-five patients subjected to brain MRI) [140]. Stroke is the leading neurological complication associated with MOPD2 syndrome. Strokes may be ischemic, hemorrhagic, or both. They are caused by progressive occlusive cerebral arteriopathy, which leads to stenosis and intracranial arterial aneurysms. This progressive arteriopathy is associated with developing compensatory capillary collaterals (smoke-like vessels) named moyamoya disease. Strokes or aneurysmal subarachnoid hemorrhages affected 51% of the reported patients who underwent brain MRI (28 out of 55) [35,130,131,135,135,138,139,140,141,142,143,144,148,151] and occurred early (median: 4.2 years old, range: 0.5–26 years). Moyamoya disease was reported in 13 out of 55 patients who underwent brain MRI [35,130,135,138,140,144,151,152]. These cerebral neurovascular anomalies worsen the functional prognosis and are a significant cause of death in patients. The consequences of both microcephaly and stroke syndrome are difficult to assess, as only 17 patients have been evaluated using neuropsychological tests. Neuropsychological assessment of these patients showed a mean full-scale intelligence quotient (FSIQ) of 65 ± 17.8 SD (range 38 to 88). No correlation could be established between intellectual abilities and the severity of microcephaly or stroke, owing to the small number of patients evaluated.

#### 4.1.4. Associated Features or Comorbidities

Heart attack with myocardial infarction caused by coronary artery disease has been reported in 3.2% of patients (4 of 123, diagnosed at 1, 15, 20, and 23 years of age) [141,151]. Insulin-resistant diabetes and dyslipidemia have been identified in almost all adult patients [150]. Lorenzo-Betancor et al. and Huang et al. reported that heterozygous carriers of *PCNT* variants could develop intra-cranial aneurysms or subarachnoid hemorrhages and must be followed up closely [131,156]. 

## 5. *CDK5RAP2*, *CEP152*, and *PLK4*: Three Emblematic PM Genes Associated with Short Stature or Chorioretinopathy or Both

### 5.1. CDK5RAP2: Phenotype–Gene Relationships

#### 5.1.1. Genetics

The *CDK5RAP2* (NM_018249.6) gene encodes CDK5 regulatory subunit associated protein 2 (CEP215), a major protein involved in PCM organization [157,158,159,160,161] (see Figure 3). As a core PCM protein, CDK5RAP2 participates in the nucleation of microtubules and the formation of mitotic spindles. Mutations in *CDK5RAP2* are a rare cause of autosomal recessive PMs. Since the identification of the first patients [4], 45 individuals from 23 families, along with 26 different biallelic variants which spread over the gene, have been reported (see synthesis in [12] and more recently [64,72]). Approximately 55% of published cases were of Pakistani origin. Of the 26 variants, 24 were loss-of-function mutations predicted to lead to the absence of a protein or a nonfunctional truncated protein. However, the effects of these variants on protein function have not been studied. 

#### 5.1.2. Growth

Short stature and MPD are associated with CDK5RAP2-PM in consanguineous families living on the Asian continent [64,162,163,164]. However, European individuals carrying compound heterozygous variants have normal heights. The position of the mutation does not seem to interfere with the phenotype. Many children with short stature carry mutations within the third, twenty-fifth, or thirtieth exon out of 38, and no variants are located in the domain of interaction with PCNT. In contrast, mutations at identical locations cause short stature in a few children but not others. The average height of the 24 individuals for whom the data are available showed a deviation of −2.8 ± 2.2 SD from the mean normal height. The following hypotheses may explain this inter-individual and geographic variability in height growth: (i) additional variants in MPD genes in consanguineous families, (ii) age of children (spontaneous correction of height post-infancy), and (iii) feeding- or nutrition-related difficulties, or both due to developmental disease or geographic or social context. 

#### 5.1.3. Brain Development and Cognition

Typically, microcephaly is detected before or at birth. As for other PMs, the kinetics of brain growth decreases with age (deviation from the mean normal OFC for the 33 patients for whom data are available was −6.6 ± 3.5 SD) in only a proportion of patients. However, a remarkable improvement in brain growth was observed after 2 years of age in others [12], suggesting that mature neurons manage to develop an efficient neuron–glial network, as evident from the normal structure of their corpus callosum and the absence of MCD. This observation is consistent with the preserved intellectual abilities of these patients, which were higher than those of patients with *ASPM* mutations. The mean value of the FSIQ of 17 assessed patients was 64.6 ± 15.3 SD, ranging from borderline intellectual functioning to mild ID [12,165,166,167,168,169,170]. Along with a simplified gyration typical of PMs, CDK5RAP2-PM has a particularity, i.e., a defect in early diencephalon development that corresponds to a “forme fruste” of holoprosencephaly characterized by the non-separation of hypothalamic nuclei along the midline [12]. This malformation has also been described in STIL-PM or MCPH7 [171,172].

#### 5.1.4. Neurosensory Impairment

Progressive sensorineural hearing loss was identified in four of seven patients from a French series after 6 years of age [12]. This loss of hearing, never observed in PMs, revealed cochlear dysplasia in six of these seven patients characterized by a small and incomplete or simplified cochlea, with only one and a half turns instead of the normal two and a half turns. This cochlear simplification, called Mondini dysplasia, is one of the hallmarks of CDK5RAP2-PM and suggests that CDK5RAP2 is crucial for ear development, as shown by its expression in the fetal cochlea. It is associated with an enlarged vestibular aqueduct and cochlear nerve hypoplasia.

Abnormal eye development is also associated with microphthalmia (a developmental disorder of one or both eyes that are abnormally small at birth) and CDK5RAP2-PM mutations. Specific chorioretinopathy, characterized by the hypo-and hyperpigmentation of the retina and reminiscent of lipofuscin deposits/accumulation in the retinal pigment epithelium, has also been observed. This feature did not impair visual acuity in the examined children [12], unlike other PMs with chorioretinopathies cited below and in Box 2.

### 5.2. CEP152: Phenotype–Gene Relationships

#### 5.2.1. Genetics

The *CEP152* gene (NM_001194998.2) encodes a centrosome protein of 152 kDa, located at the proximal end of the parent centriole, that recruits PLK4 to build a new procentriole in the S phase [173,174,175,176,177,178,179] (see Figure 3). Mutations in *CEP152* are a rare cause of autosomal recessive PMs. Since the first patients were identified [8,9], 14 individuals from 9 families with 12 biallelic variants have been reported [8,9,93,180]. Five of the nine variants were loss-of-function mutations predicted to lead to the absence of a protein or a non-functional truncated protein. A founder haplotype was identified in seven Turkish individuals via a recurrent homozygous splice donor-site mutation in intron 4 (c.261+1G>C). This variant led to the formation of four different aberrant transcripts likely to cause a loss of protein function; however, the partial functional activity of one mutant protein, Val86_Asn87del, cannot be excluded [8].

#### 5.2.2. Growth

*CEP152* mutations may cause two distinct PM phenotypes, i.e., the MCPH phenotype or the MPD phenotype resembling Seckel syndrome. The MCPH phenotype was observed in three Canadian individuals from different families of Acadian descent, with normal stature carrying the p.Q265P protein variant. The MPD phenotype resembling Seckel syndrome was observed in eight individuals from four distinct Turkish, South African, or Chinese families, with a deviation of −5.4 ± 1.9 SD from the mean normal height for a mean age of 9.3 ± 5.4 years [8,180]. Height was not indicated for the two Pakistani individuals [93]. These individuals with CEP152-MPD also exhibited dysmorphic features with a high nasal bridge and beaked nose, i.e., “bird-head”, a fifth finger clinodactyly (curved finger deviated in a radioulnar or mediolateral direction may overlap other fingers), tooth agenesis, and retrognathia (abnormal positioning of the mandible). However, unlike individuals with PCNT-related phenotype, the subgroup with CEP152-MPD did not exhibit proportionate dwarfism (height and OFC were not equally reduced), as expected in patients with Seckel syndrome.

#### 5.2.3. Brain Development and Cognition

In these 13 individuals, the reduction in brain size was relatively more pronounced than in body size and reached a deviation of −7.7 ± 2.9 SD from the mean normal brain size at an average age of 9.38 ± 6.4 years. Gyral simplification was associated with microcephaly in six cases. Three individuals with MCPH-like phenotype seemed to have preserved cognitive functions as they could read and attend regular classes with or without modifications until 11 years of age. One individual benefited from a neuropsychological assessment showing borderline intellectual functioning evident in visual motor skills. Behavioral disorders, such as tantrums, tics, and obsessive/compulsive traits, were reported in three patients [9]. Intellectual disability appeared to be higher in patients with MPD, although there have been no real neuropsychological assessments of these individuals [8,93].

#### 5.2.4. Neurosensory Impairment

No neurosensory impairment has been reported in individuals bearing mutations in the *CEP152* gene.

### 5.3. PLK4: Phenotype–Gene Relationships

#### 5.3.1. Genetics

The *PLK4* gene (NM_001190799) encodes the Polo-like Kinase 4, the master regulator of centriole duplication, which is activated upon phosphorylation and recruits STIL and SAS6 proteins at the proximal end of the parent centriole, thereby initiating the assembly of the procentriole [21,177,179,180,181,182] (see Figure 3). Mutations in *PLK4* are an exceedingly rare cause of autosomal recessive PMs. Since the identification of the first patients [46], 15 individuals from seven families carrying five different biallelic variants have been reported [46,48,181,182,183]. A recurrent homozygous mutation (c.1299_1303delAAAG; p. Phe433Leufs*6) has been reported in four families from diverse geographical regions (Madagascar, Iran, Pakistan, and Equatorial Guinea). Levels of functional PLK4 transcripts were reduced to 25% of healthy control levels in individuals with such mutations [46]. The c.2811–5G>C variant created a new splice acceptor site that led to the retention of 4 bp from the intron 15 sequence in PLK4 mRNA, resulting in the premature truncation of the protein at its C terminus and disruption of the terminal Polo-box domain [46]. The c.31-3 A4G substitution disrupts the splicing of the first intron and leads to the transfer of 63 nucleotides from the acceptor site of intron 1 to the mature mRNA. This results in a frameshift and premature translation termination (=, Asp11Profs*14). Four mildly affected individuals have been reported to carry a novel missense variant, c.881 T>G, with no effect on OFC and cognitive functions in the homozygous state, and a hemizygous deletion spanning complete *PLK4* and *MFSD8* genes and exon 1 of *ABHD18* [184].

#### 5.3.2. Growth

All 15 patients exhibited an MPD phenotype with a deviation of −6.5 ± 1.3 SD from the mean normal height at an average age of 6.6 ± 6 years. Unlike individuals with PCNT-related phenotype, individuals carrying *PLK4* mutations did not exhibit proportionate dwarfism (height and OFC not reduced equally) as expected in Seckel syndrome.

#### 5.3.3. Brain Development and Cognition

Similar to mutations in *CEP152*, mutations in the *PLK4* gene alter brain growth more than body growth, as the reduction in OFC expression is more pronounced (−11.7 ± 2.3 SD) than in height. The gyral pattern of the brain is extremely simplified [182] and represents what is called microlissencephaly. Interhemispheric arachnoid cysts have been reported in two cases in unrelated patients. Neuronal heterotopia was observed in three of seven patients who underwent brain imaging [46,181,182,183]. No cases of epilepsy have been reported to date. The development of these patients was severely delayed (developmental quotient 21 [181], normal range for the general population: 80–120). Most patients were unable to sit unaided [46] and to speak, which indicates a severe-to-profound intellectual disability.

#### 5.3.4. Neurosensory Impairment

In the brain, the development of the optic vesicle was insufficient based on age (microphthalmia, microcornea, and optic nerve hypoplasia) regardless of the mutation [46,48,181,182], abnormal with a persistent hyperplastic primary vitreous at the origin of retinal detachment and blindness in one patient [182]. Deafness has been reported in three unrelated patients carrying different mutations [46,182].

## 6. Emblematic PM Genes Encode Centrosome or Spindle Pole Proteins

The centrosome is an organelle without a membrane, composed of two centrioles and a surrounding PCM, which considerably increases its size and capacity to nucleate microtubules during mitosis (see reviews detailing recent advances in centrosome structure and function [24,185]). Thus, the centrosome and minus end of microtubules, mainly nucleated from centrioles during mitosis, form the mitotic spindle pole [24,186,187,188,189]. Deficits in one or the other centrosome or spindle pole protein could severely affect brain development, particularly during the development of the cerebral cortex, mainly affecting mitotic spindle organization, stability, and orientation (see excellent and recent reviews on these topics [17,20,21,190,191]). 

During mitosis, ASPM and WDR62 are located at the minus end of microtubules at the centrosome, PCNT and CDK5RAP2 at the PCM, whereas CEP152 and PLK4 are centriolar proteins (Figure 3). Despite most PM genes encoding mitotic apparatus proteins, exceptions exist, in particular, *ZNF335* and *PHC1* related to transcription/chromatin remodeling processes, *NCAPD2/3* and *NCAPH* involved in chromosome condensation, or a few others involved in DNA damage response (see Appendix A Table A1).

## 7. Unresolved Issues and Clinical Pitfalls

### 7.1. Mutations in Emblematic PM Genes Playing a Role *in Spindle Pole Structure and Function Are Responsible for Different Diseases with Partial Overlap*

Researchers and physicians have long wondered whether the absence of one or more of these spindle pole proteins invariably causes a single disease, i.e., “PM”. The preceding paragraphs show that it is not the case. The absence of specific PM proteins will not lead to similar consequences on brain and neurosensory development or the size of individuals. Mutations in *ASPM, WDR62,* and *DYNC1H1* genes only affect brain size and structure, whereas mutations in *CDK5RAP2*, *CEP152*, and *PLK4* affect brain size more than body size. Only the mutations in *PCNT* equally reduced brain and body size. In addition, mutations in *CDK5RAP2* and *PLK4* cause neurosensory impairments (see Figure S1 and Appendix A Table A1). The effects of mutations in one or another PM gene on brain structure and function, i.e., on the intellectual abilities of patients, will differ.

The functional impairment of microtubule minus-end-targeting proteins ASPM and WDR62, and the main minus-end-directed motors, DYNC1H1, affects brain growth and structure, whereas that of centriolar or PCM proteins PLK4, PCNT, and CDK5RAP2 affects both brain and body size, but to different degrees. Together, these ubiquitously expressed proteins are a part of the microtubule-organizing center, the centrosome, or the spindle pole. Many questions remain unanswered. First, a loss of one of these PM proteins out of a thousand results in a collapse of the centrosome structure. Is there no redundancy? Does the absence of this specific PM protein explain cellular and clinical phenotypes? Alternatively, does the loss of interactions between these PM proteins and their partners explain these defects? Second, how are a few of these mitotic apparatus proteins essential for the growth of all organs and others only for brain growth?

In summary, although they participate in the same function, organization, and stability of the mitotic spindle, their role is not redundant at the organ or organismal scale. The absence or presence of a truncated form of one PM protein cannot be compensated for by the existence or the over-representation of others. 

### 7.2. Why Is the Brain Relatively More Vulnerable than Other Organs?

This question has fascinated researchers for many years and remains unsolved. For example, centrosome proteins are expressed ubiquitously. How to explain then that mutations in PM genes regulating the function of centrosomes will have relatively more consequences on the development of the brain than that of other organs? Three alternative hypotheses can be proposed. The first hypothesis would be related to the short time window of neurogenesis. Indeed, neural progenitors do not proliferate throughout life, unlike progenitors from other organs. Neuron production is restricted to a short period via neurogenesis, which occurs between 6 weeks post-conception (WPC) and 22–24 WPC in humans. The final number of neurons generated during neurogenesis is fixed by the end of the second trimester of pregnancy. No compensation is possible later during brain development. The second hypothesis could be that different isoforms are expressed in a tissue- or organ-specific manner in distinct tissues/organs. It has been shown that a few PM genes (*ASPM*, *MCPH1*, *Cdk5rap2*, and *Nin*) encode different protein isoforms in humans and mice. These isoforms are relatively more expressed in fetal than in adult tissues/cells and may have different localization patterns and functions throughout life [76,192,193,194]. For example, alternative splicing is responsible for a change in the localization of ninein from centrosome in neural progenitors to non-centrosome sites in neurons [21,194]. The third hypothesis could be related to tissue-specific characteristics. For example, it is to note that the embryonic brain, which is a neuroepithelium and all epithelia (skin, kidney, retina, hair cells of the organ of Corti, and secretory cells from the pituitary gland or pancreas) are polarized tissues and also the most affected by mutations in PM genes (see Figure S1 and Appendix A Table A1). Among polarized tissues, the neuroepithelium and the neurosensory epithelium do not proliferate throughout life. The question remains whether such vulnerability is due to the morphological characteristics of these epithelial cells or to different levels of polarity, among other possibilities [195]. 

### 7.3. Do Single Nucleotide Polymorphisms, Not Pathogenic, or Heterozygous Pathogenic Variants in PM Genes Affect Cognitive Functions, Brain Size, or Both?

The genes coding for PM proteins evolved rapidly between ancestral primates and humans (for a review, see [196]. New haplotypes (benign sequence variations or single nucleotide polymorphisms (SNPs)) in PM genes emerged during human evolution as a result of a positive selection that may contribute to brain enlargement [197,198,199]. Researchers investigated possible correlations between common PM-related SNPs and variation in brain volume or intelligence. No correlation has been identified between ancestor SNPs in *ASPM* and *MCPH1* and (i) IQ scores, (ii) head circumference, or (iii) brain volume assessed by MRI [200,201,202]. Sex-specific associations have been found between common non-exonic SNPs in *ASPM*, *MCPH1,* and *CDK5RAP2* genes and brain volume or cortical surface area in two different series, a Norwegian and a North American series of healthy controls and patients with mental illness or dementia, respectively [203]. 

The consequences on brain size or cognition of pathogenic variants carried in a homozygous state by the parents of patients remain unknown. Intriguingly, it may influence brain size, as inferred from the OFC measurements of parents, relatively more frequently below the normal range than in the general population (personal observation). Nevertheless, it does not seem to affect the cognitive abilities of these individuals, as indicated by their academic course and economic and social position.

### 7.4. Do Mutations in One or More PM Genes Allow the Establishment of Prognosis?

Although it is and will always be extremely difficult for physicians to determine long-term prognosis in these patients, this review shows that there are specificities for each of these PMs and that a few PM types are relatively more severe than others. In addition to brain volume reduction, PM severity is based on the following factors: (i) risk of a life-threatening situation (stroke), (ii) associated comorbidities (neurosensory impairment and epilepsy), or (iii) degree of intellectual disability. 

Molecular diagnosis enables prognosis to be specified. *PCNT* mutations are the cause of premature death when aneurysms rupture. Although they represent one of the rarest forms of PM, mutations in *PLK4* appear to affect intellectual abilities more severely than mutations in other PM genes. Efforts must continue to identify PMs that have the severest impact on the cognitive functions of patients, which will require more patients to be assessed intellectually and additional anatomical-functional and genotype-functional correlations to be examined. 

However, identifying causal mutations is insufficient for establishing a prognosis. Other factors come into play and modulate the effect of mutations on brain growth and cognitive functions. Variability exists among affected patients and siblings carrying mutations in the same gene. These factors may be genetic, and the possibility of digenism in primitive microcephaly is beginning to emerge [49,66]. However, they may also be epigenetic. Such mutations or the presence of other likely benign mutations might together modulate regulatory components or chromatin modifiers, thus increasing the risk of neurodevelopmental disorders that worsen patient prognosis. Finally, environmental factors (infectious or toxic) may affect brain development during neurogenesis. None of these additional hypothetical factors will be easy to identify without carefully following the underlying factors in each pregnant woman, sequencing the whole genome, and exploring the epigenetic pathways for each patient. 

### 7.5. Understanding What Occurred during Neurogenesis in the Brain of Each Individual Affected by PM

Several knockdown animal models have facilitated deciphering mechanisms underlying these emblematic PMs. These mechanisms are based mainly on premature neuronal differentiation and the death of neural progenitors [81,204,205,206,207,208]. However, the absence of a PM protein may not produce the same alterations as a mutated/truncated form of this protein in developing organs, which needs to be considered to elucidate what occurs in the brains of patients during development. Brain organoids are obtained by reprogramming patient cells into induced pluripotent stem cells (IPSCs) and then differentiating them into neural progenitors used to study defects caused by PM mutations during brain development in the genomic context of each patient. Such approaches were used initially to investigate the mechanisms underlying PM and associated defects. Brain organoids have become a powerful tool to model human microcephaly [163]. Generating a mutation or deletion in a PM gene in the IPSCs of healthy controls and differentiating them into brain organoids is the most common method to dissect the role of a gene in human brain development [81], but not to identify altered pathways in patients’ brains. Another approach, centered on their progenitors and neurons, will be necessary to develop personalized medicine for patients.

### 7.6. Identifying the Temporal Window during Which Defects Occur and during Which Intervention Will Be Possible to Improve the Conditions of Patients

The specificity of the brain, compared with other organs, results from the fact that neural progenitors do not proliferate throughout life. Neurons are produced between 6 weeks post-conception (WPC) and 22–24 WPC in humans. Ultrasound detects neurogenesis impairments with a delay of several weeks, and despite a quick molecular diagnosis, within days or a few weeks, it is generally late in pregnancy or postnatally, and it is also too late to act. However, no compensatory mechanism has been considered. Owing to the non-feasibility of restarting neuronal production after the end of neurogenesis, and hence, enlarging the brain of individuals affected by PM, the only medical option is early intervention in childhood to improve the skills of affected children. This program includes physical and occupational therapies, speech therapy, and psychological support. Antiepileptic drugs are often required in children with seizures.

Physicians, patients, and their families have different expectations. To accept that everything is written before or at birth for the remainder of life is hard. After identifying the underlying mechanisms involved, the question arises of when and how to influence the trajectory of neurons in these individuals to make them relatively more efficient. Physicians and researchers must work together to reflect on this issue, find a way to specifically answer the needs of each patient, and propose a personalized intervention. 

## 8. Conclusions

Since the discovery of PM genes in the early 2000s, our knowledge of neurodevelopmental diseases has increased considerably. Genetics uncovered genes encoding components of the mitotic spindle implicated in primary microcephaly. Animal models have revealed the underlying mechanisms and consequences of the absence of one of the PM proteins in brain development. However, the impact of mutated/truncated PM proteins on brain growth, brain structure, and cognitive functioning in affected patients remains under investigation, making long-term prognosis and early intervention difficult. Nevertheless, this review, combining clinical, cognitive, and imaging data of reported patients with PM, highlights that not all primary microcephalies are similar. Dysfunction in microtubule minus-end proteins at the spindle pole (ASPM and WDR62) or minus-end motor proteins (DYNC1H1) only affects brain growth and structure, whereas dysfunction in centriolar proteins (PLK4 and CEP152) or pericentriolar matrix proteins (PCNT and CDK5RAP2) affects both brain and body growth. Therefore, in addition to conducting further research on impaired pathways in the developing brains of patients using brain organoids, physicians need to explore how the brains of these patients function by assessing their cognitive abilities and thus propose innovative interventions. 

## Data Availability

Not applicable.

## Supplementary Materials

The following supporting information can be downloaded at: https://www.mdpi.com/article/10.3390/cells12131807/s1, Figure S1: Affected organs related to mutations in PM and MPD genes (created with BioRender.com, accessed on 5 April 2023).

## Appendix A

**Table A1:** **Main genes (proteins) mutated in Primary Microcephaly (PM) and Microcephalic Primary Dwarfism (MPD), their functions, functional partners and affected organs.** We do not aim in this table to provide an exhaustive list of the genes mutated in PM, MPD and PM with neurosensory disorders, which were recently documented elsewhere [15,21,191,209]. Note that we report here only the partners that are involved in the same functional pathway than that of the PM/MPD proteins.

Locus	Gene/Protein	Subcellular Location	Main Molecular Functions	Functional Partners Involved in the Same Pathway	Syndrome Related to Mutations	Affected Organs	References
MCPH1	*MCPH1*/ MICROCEPHALIN	Nucleus, mitochondria	Chromosome condensation, DNA replication, DNA damage response	CDC27, H2AX upon DNA damage	PM/MPD (AR)	Brain, skeleton	[2,210,211,212,213,214,215]
MCPH2	*WD62*/WDR62	Minus end of microtubules	Neuronal proliferation and migration	ASPM, AURORA A, CEP152, CEP63, CDK5RAP2	PM + MCD (AR)	Brain	[3,10,11,216,217]
MCPH3	*CDK5RAP2*/ CDK5RAP2	PCM	Microtubule nucleation, centriole disengagement after duplication	PCNT, GAMMA TUBULIN, WDR62, CEP152, CEP63	PM (AR)	Brain, eye, ear	[4,162,216,218,219,220,221]
MCPH4	*CASC5*/KLN1	Kinetochore	Attachment of centromeres to spindle microtubules, spindle-assembly checkpoint signaling	BUB1, BUB1B	PM (AR)	Brain	[222,223,224,225,226]
MCPH5	*ASPM*/ASPM	Minus end of microtubules	Spindle pole organization, microtubule dynamics at spindle pole	KATNA1, KATNB1, WDR62, CIT	PM (AR)	Brain	[6,56,76,217]
MCPH6 & SCKL4	*CENPJ (CPAP)*/ CENPJ (CPAP)	Centriole	Centriole duplication/biogenesis	CEP152, STIL, SAS6, GAMMA TUBULIN, CEP135, microtubules	PM/MPD (AR)	Brain, skeleton	[4,221,227,228,229,230,231]
MCPH7	*STIL*/STIL	Centriole cartwheel	Centriole duplication/ biogenesis, primary cilium	PLK4, CENPJ/CPAP, SAS6	PM/MPD (AR)	Brain	[229,232,233,234,235,236]
MCPH8	*CEP135*/CEP135	Centriole	Centriole biogenesis & cohesion	CEP152, CENPJ/CPAP, SAS6, GAMMA TUBULIN, Microtubules	PM/MPD (AR)	Brain	[231,237,238,239,240]
MCPH9 & SCKL5	*CEP152*/CEP152	Centriole	Centriole duplication/ Biogenesis	PLK4, CENPJ/CPAP, WDR62, CEP192, CEP63, CEP135, CDK5RAP2	PM/MPD (AR)	Brain, skeleton, teeth	[8,173,174,216,221,228,231,241]
MCPH10	*ZNF332*/ZNF332	Nucleus	Transcription, neural progenitor proliferation	CCAR2, EMSY, ASCL2	PM (AR)	Brain, skeleton, eye, ear	[242,243]
MCPH11	*PHC1*/PHC1	Nucleus	Chromatin remodeling	PRC1 complex	PM/MPD (AR)	Brain	[244]
MCPH12	*CDK6*/CDK6	Nucleus, centrosome	Cell cycle control (G1/S transition)	CYCLIN D	PM (AR)	Brain	[245,246]
MCPH13	*CENPE*/CENPE	Plus-ends of microtubules, kinesin-like motor protein	Microtubule attachment at the kinetochore, chromosome congression	CENPF, BUB1B	PM/MPD (AR)	Brain, skeleton, heart	[247,248,249,250]
MCPH14	*SAS6*/SAS6	Centriole cartwheel	Centriole duplication/ Biogenesis	CENPJ/CPAP, STIL, CEP135, CEP152	PM (AR)	Brain	[174,229,231,251,252,253]
MCPH15	*MSFD2A*/ MSFD2A	Cell membrane	Blood-brain barrier formation		PM (AR)	Brain	[254,255,256]
MCPH16	*ANKLE2*/ANKLE2	Endoplasmic reticulum	Nuclear envelope reassembly	BAF/BANF1	PM/MPD (AR)	Brain, eye, skin, bone marrow, skeleton, heart	[148,257,258]
MCPH17	*CIT*/CIT	Midbody	Cytokinesis	ASPM, KIF14	PM/MPD (AR)	Brain	[259,260,261,262,263]
MCPH18	*WDFY3*/WDFY3	Nucleus	Autophagy	ATG5	PM (AD)	Brain	[264,265]
MCPH19	*COPB2*/COPB2	Golgi, cytoplasmic vesicle	ER-to-Golgi and Golgi-to-ER transport. Coatomer complex protein	alpha, beta, gamma, delta, epsilon and zeta COP-subunits	PM (AR)	Brain, skeleton	[266]
MCPH20	*KIF14*/KIF14	Mitotic spindle, midbody	Cell cycle progression, cytokinesis	CIT (AR)	PM/MPD (AR)	Brain, +/− kidney and eye	[262,267,268,269]
MCPH21	*NCAPD2*/NCAPD2	Nucleus, chromosomes	Mitotic chromosome condensation/integrity	Component of the condensing complex NCAPD3/H/G	MPD (AR)	Brain	[270,271]
MCPH22	*NCAPD3*/NCAPD3	Nucleus, chromosomes	Mitotic chromosome condensation/integrity	Component of the condensing complex NCAPD2/H/G	PM/MPD (AR)	Brain	[270,271]
MCPH23	*NCAPH*/NCAPH	Nucleus, chromosomes	Mitotic chromosome condensation/integrity	Component of the condensing complex NCAPD2/D3/G	PM (AR)	Brain	[270,271]
MCPH24	*NUP37*/NUP37	Nucleus, nuclear pore complex	Kinetochore microtubule attachment, mitotic progression	Component of the nuclear pore complex (NUP)	PM (AR)	Brain	[272,273]
MCPH25	*MAP11*/MAP11	Mitotic spindle, midbody	Mitotic spindle dynamics, cytokinesis	TUBA1A	PM (AR)	Brain	[274]
MCPH26	*LMNB1*/LMNB1	Nucleus, inner nuclear membrane	Nuclear shape, spindle assembly	Lamin-associated polypeptides	PM (AD)	Brain	[275,276,277,278]
MCPH27	*LMNB2*/LMNB2	Nucleus, inner nuclear membrane	Nuclear shape, spindle assembly	Lamin-associated polypeptides	PM (AD)	Brain	[275,276,277,278]
MCPH28	*RRP7A*/RRP7A	Nucleus, cilium, microtubules	Ribosome biogenesis, cilium resorption	Small subunits of the processosome	PM (AR)	Brain	[279,280]
MCPH29	*PDCD6IP*/ PDCD6IP	Cytoplasmic vesicle membrane	Cytokinesis, apoptosis	ESCRT-III components	PM (AR)	Brain	[281]
MCPH30	*BUB1*/BUB1	Chromosomes, kinetochore	spindle-assembly checkpoint signaling, chromosome segregation	MAD1L1, BUB3, CASC5/KNL1, CENPE	PM/MPD (AR)	Brain, skeleton, heart	[282,283,284,285]
MOPD2	*PCNT*/PCNT	PCM	PCM formation and centrosome cohesion	GAMMA TUBULIN, CDK5RAP2	MPD, MOPD2 and Seckel syndromes (AR)	Brain, cerebral arteries, heart, kidney, bone marrow, skin, pituitary gland, pancreas, skeleton, teeth	[35,36,286,287,288,289]
SCKL1	*ATR*/ATR	Nucleus, chromosomes	DNA damage response	ATRIP, RAD17	MPD (AR)	Brain, skeleton, bone marrow, teeth	[36,290,291,292]
SCKL2	*RBBP8 (CTIP)*/RBBP8	Nucleus, chromosomes	Double-stand break repair, homologous recombination	BRCA1, MRN complex, RAD50	MPD (AR)	Brain, skeleton, eye, teeth	[293,294,295,296]
SCKL6	*CEP63*/CEP63	Mother Centriole	Centriole integrity/assembly	CEP152, CDK5RAP2, WDR62	MPD (AR)	Brain	[216,297,298]
SCKL7	*NIN*/NINEIN	Sub-distal appendage of mother centriole	Microtubule anchoring at mother centriole	CCDC120	MPD (AR)	Brain, pituitary gland, skeleton	[299,300,301]
SCKL8	*DNA2*/DNA2	Nucleus, mitochondria	DNA replication/repair	BLM	MPD (AR)	Brain	[302,303,304,305]
SCKL9	*TRAIP*/TRAIP	Nucleus	Replication stress response	PCNA	MPD (AR)	Brain, gonads, skeleton, lung, kidney	[306,307,308,309]
SCKL10	*NSMCE2*/ NSMCE2	Nucleus	DNA double-strand break repair–homologous recombination	Component of the SMC5-SMC6 complex	MPD ‘AR)	Brain, eye, pancreas, gonads	[310,311,312]
MCCRP1	*TUBGCP6*/ TUBGCP6	PCM/MTOC	Microtubule nucleation	TUGCP2/3/4/5, GAMMA TUBULIN	PM + neurosensory disorder (AR)	Brain, eye	[46,313]
MCCRP2	*PLK4*/PLK4	Centriole	Centriole duplication/ Biogenesis	CEP152, CEP192, STIL	MPD + neurosensory disorder (AR)	Brain, eye, ear, skin	[46,174,209,237,314,315]
MCCRP3	*TUBGCP4*/ TUBGCP4	PCM/MTOC	Microtubule nucleation	TUGCP2/3/4/5, GAMMA TUBULIN	PM + neurosensory disorder (AR)	Brain, eye	[47,316]
MCLRM	*KIF11*/KIF11	Plus end of microtubules	Bipolar spindle establishment	Microtubules	PM + neurosensory disorder (AD)	Brain, eye	[317,318]
CDCBM/SMELED	DYNC1H1	Microtubules	Transporter of cargo towards the minus ends of microtubules	DYNC1LI1/2, DYNACTIN, LIS1, NDEL1	PM + MCD (AD)	Brain, muscle	[100,319,320,321]
Bloom (BLM) syndrome	*RECQL3*/RECQL3	Nucleus	DNA replication/repair	RAD51, FANCD2, DNA2	MPD (AR)	Brain, skin, pituitary gland, bone marrow, immune system, gonads, pancreas	[322,323,324,325]
LIG4 syndrome	*LIG4*/LIG4	Nucleus	DNA repair	XRCC4/5/6, NHEJ1/XLF	MPD (AR)	Brain, immune system, skin	[39,326,327,328]
SSMED	*XRCC4*/XRCC4	Nucleus	DNA repair	XRCC5/6, LIG4,	MPD (AR)	Brain, eye, ear, pituitary gland, gonads, pancreas, immune system, kidney, +/− heart	[40,328,329]
Meyer Gorlin syndrome 1	*ORC1*/ORC1	Nucleus	DNA replication	ORC2/3/4/5/6, CDC6	MPD (AR)	Brain, skeleton	[37,330,331]
Meyer Gorlin syndrome 2	*ORC4*/ORC4	Nucleus	DNA replication	ORC1/2/3/5/6, CDC6	MPD (AR)	Brain, skeleton	[241]
Meyer Gorlin syndrome 3	*ORC6*/ORC6	Nucleus	DNA replication	ORC1/2/3/4/5, CDC6	MPD (AR)	Brain, skeleton	[37]
Meyer Gorlin syndrome 4	*CDT1*/CDT1	Nucleus, chromosomes, kinetochore	DNA replication, mitosis	CDC6, PCNA, GMMN	MPD (AR)	Brain, skeleton	[37,241,332,333]
Meyer Gorlin syndrome 5	*CDC6*/CDC6	Nucleus	DNA replication	ORC1, CDT1, PCNA	MPD (AR)	Brain, skeleton, teeth	[37,334,335]
Meyer Gorlin syndrome 6	*GMNN*/GMNN	Nucleus	DNA replication	CDT1	MPD (AD)	Brain, skeleton, ear	[336,337]

CDCBM: Cortical dysplasia, complex brain malformation; ER: Endoplasmic reticulum; ESCRT III: endosomal sorting required for transport complex III; MCCRP: MicroCephaly and ChorioRetinoPathy; MCD: malformation of cortical development; MCLRM: microcephaly with or without chorioretinopathy, lymphedema or impaired intellectual development; MOPD2: microcephalic osteodysplastic primordial dwarfism; PCM: pericentriolar material; MPD: microcephalic primordial dwarfism; SMALED: Spinal muscular atrophy, lower extremity predominant; SSMED: short stature, microcephaly, endocrine dysfunction.
