# Supplementary material for: Genetic Primary Microcephalies: When Centrosome Dysfunction Dictates Brain and Body Size
Source: Cells. 2023 Jul 7;12(13):1807. doi: 10.3390/cells12131807 (PMC10340463; doi:10.3390/cells12131807)
Supplement: Supplementary file 1 [file cells-12-01807-s001.zip › cells-2360591-supplementary.pdf]

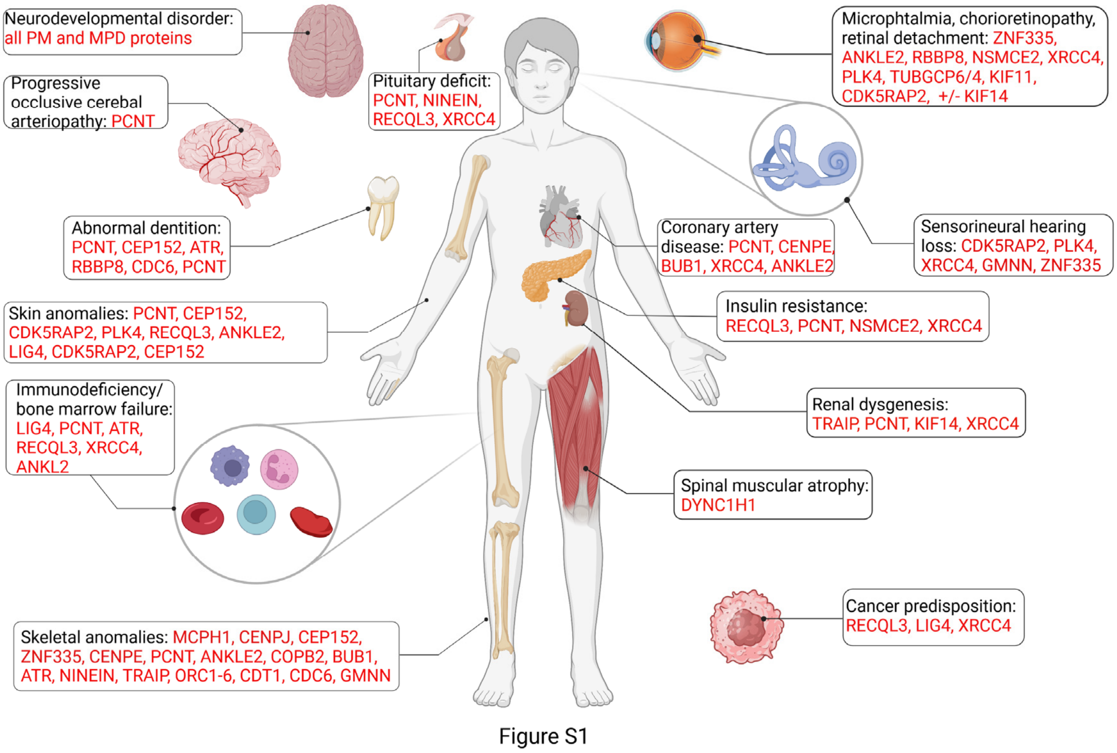

Figure S1: Affected organs related to mutations in PM and MPD genes (created with BioRender.com)
